# Predictive Value of CRIB II Score, Hematological Markers and Maternal-Fetal Risk Factors for Mortality in Neonatal Sepsis

**DOI:** 10.3390/children12121578

**Published:** 2025-11-21

**Authors:** Bugis Mardina Lubis, Cindy Clarissa Sirait, Ika Citra Dewi Tanjung, Arlinda Sari Wahyuni, Ayodhia Pitaloka Pasaribu

**Affiliations:** 1 Department of Pediatrics, Faculty of Medicine, Universitas Sumatera Utara, Medan 20155, Indonesia; 2 Department of Community Medicine, Universitas Sumatera Utara, Medan 20155, Indonesia

**Keywords:** sepsis/mortality, infant, newborn, Prognosis, biomarkers, blood, infant mortality

## Abstract

Background: Neonatal sepsis is a leading cause of neonatal morbidity and mortality, particularly in low- and middle-income countries. Accurate early prediction of mortality is essential to improve clinical outcomes. The Clinical Risk Index for Babies II (CRIB II) and hematological indices such as the platelet-to-lymphocyte ratio (PLR) have emerged as potential prognostic tools, but their performance in septic neonates has not been fully established. Methods: A retrospective observational study was conducted on 80 neonates diagnosed with sepsis at a tertiary referral hospital. Demographic, maternal, and laboratory data—including CRIB II, neutrophil-to-lymphocyte ratio (NLR), PLR, mean platelet volume (MPV), and red-cell distribution width (RDW)—were analyzed. Receiver operating characteristic (ROC) curves were used to determine optimal cutoff values, and multivariate logistic regression identified independent predictors of mortality. Statistical significance was set at *p* < 0.05. Results: Among the 80 neonates, 40 (50%) did not survive. Non-survivors had significantly higher CRIB II scores (median 5.0 [1.0–13.0]) than survivors (2.0 [0–7.0]) (*p* < 0.001). PLR and MPV were also elevated in non-survivors (PLR: 88.5 vs. 51.8, *p* < 0.001; MPV: 10.5 vs. 9.5, *p* < 0.001). ROC analysis demonstrated strong predictive accuracy for mortality: CRIB II (AUC = 0.835; cutoff > 3.5; sensitivity 82.5%; specificity 78.0%) and PLR (AUC = 0.757; cutoff > 81.33; sensitivity 80.0%; specificity 70.0%). Multivariate analysis confirmed three independent predictors—PROM > 24 h (OR = 12.23; 95% CI: 2.05–73.11), PLR > 81.33 (OR = 91.02; 95% CI: 7.00–1184.29), and CRIB II > 3.5 (OR = 101.37; 95% CI: 4.50–2284.47). Conclusions: CRIB II and PLR are reliable, independent predictors of mortality in neonatal sepsis. Their integration with maternal factors such as prolonged rupture of membranes may improve early risk stratification and guide targeted interventions, particularly in resource-limited neonatal care settings.

## 1. Introduction

Neonatal sepsis remains a major contributor to neonatal morbidity and mortality worldwide, particularly in low- and middle-income countries where diagnostic and therapeutic resources may be limited [1,2]. The clinical presentation of sepsis in neonates is often subtle and nonspecific, making early diagnosis and risk stratification a persistent challenge. Delays in identifying high-risk neonates can result in progression to septic shock, multi-organ failure, and death [1].

Globally, an estimated 1.7 million neonates develop sepsis annually, with case-fatality rates ranging from 11% to 40%, depending on gestational age, organism type, and quality of care [2]. In Indonesia, neonatal sepsis contributes substantially to neonatal mortality, with national surveys reporting an incidence of 15–20 cases per 1000 live births [3].

The clinical manifestations of neonatal sepsis are often subtle and nonspecific—such as temperature instability, feeding difficulty, or respiratory distress—making early recognition and risk stratification difficult [4]. Distinguishing between early-onset sepsis (EOS), which occurs within 72 h of birth and is typically related to maternal or intrapartum infection, and late-onset sepsis (LOS), which develops after 72 h and is commonly hospital-acquired, is crucial because their etiologies and outcomes differ [5,6]. However, both forms share overlapping pathophysiologic pathways involving systemic inflammation, endothelial dysfunction, and multi-organ compromise [4].

To improve prognostication, clinical severity scores and laboratory biomarkers have been proposed as adjunctive tools in neonatal critical care. One commonly used prognostic score is the Clinical Risk Index for Babies II (CRIB II), which integrates parameters such as birth weight, gestational age, sex, admission temperature, and base excess. CRIB II was developed to reduce bias from early treatment and to provide a simplified, recalibrated version of the original CRIB score [7]. Several studies have validated the utility of CRIB II in predicting overall neonatal mortality; for example, neonates with CRIB II scores above 7 have been associated with significantly higher mortality [8]. In comparative analyses, CRIB II has demonstrated better discriminative ability than gestational age or birth weight alone in certain preterm subgroups [9,10]. However, the predictive performance of CRIB II specifically among neonates with sepsis has not been fully elucidated. In studies comparing prognostic scores, CRIB II has been evaluated alongside the neonatal Sequential Organ Failure Assessment (nSOFA) and SNAPPE-II scores for mortality prediction, but specific investigations focusing on septic subpopulations remain limited [10].

In parallel with clinical severity scoring systems, hematological parameters derived from routine complete blood counts represent attractive prognostic candidates, as they are inexpensive, universally accessible, and rapidly obtainable. Parameters such as total leukocyte count, absolute neutrophil and lymphocyte counts, platelet count, mean platelet volume (MPV), and derived indices—including the neutrophil-to-lymphocyte ratio (NLR) and platelet-to-lymphocyte ratio (PLR)—have been investigated in the context of neonatal sepsis [11,12]. A retrospective analysis of 265 neonates with sepsis reported significantly higher median NLR values in affected neonates compared with controls, although its discriminative performance was modest (area under the curve [AUC] ≈ 0.57) [11]. More recently, a cohort study demonstrated that alterations in leukocyte count, hematocrit, and thrombocytopenia were frequently observed among neonates with sepsis, supporting their potential role as early prognostic markers. Nevertheless, most available studies evaluating hematological predictors have focused on their diagnostic utility—distinguishing septic from non-septic neonates—rather than their prognostic significance in predicting mortality, and many have relied primarily on univariate analyses [12,13,14].

Beyond neonatal factors, maternal and perinatal conditions play a crucial role in determining the risk and outcomes of neonatal sepsis. Conditions such as prolonged rupture of membranes (PROM), intrapartum fever or chorioamnionitis, inadequate antenatal care, and maternal infections may influence the neonatal inflammatory milieu or predispose the infant to severe infection. Some studies have shown associations between maternal infection and neonatal late-onset sepsis, while others have explored correlations between biomarkers and PROM-related sepsis. However, the integration of maternal and perinatal variables into prognostic models for neonatal sepsis mortality has been less systematically addressed [13,14,15,16].

Given these gaps, this study aims to enhance prognostic stratification among septic neonates by combining clinical severity scoring (CRIB II) and hematologic parameters within a multivariable framework that also accounts for maternal and perinatal covariates. Such an approach may assist clinicians in the early identification of high-risk infants, optimize resource allocation, and inform timely therapeutic decisions.

## 2. Materials and Methods

### 2.1. Study Design and Setting

This study employed a retrospective cohort design to evaluate the association between the CRIB II score, hematological parameters, and neonatal mortality among infants with sepsis. Data were retrospectively retrieved from medical records of neonates admitted between January 2024 and September 2025 to the Neonatal Intensive Care Unit (NICU) of a tertiary referral teaching hospital in Medan, North Sumatra, Indonesia. The NICU provides advanced perinatal and intensive care services and serves as a regional referral center for high-risk neonates across Northern Sumatra.

### 2.2. Participants and Sampling

A total of 80 neonates diagnosed with sepsis were included in this retrospective study. Neonatal sepsis was defined according to national and World Health Organization (WHO) neonatal infection criteria, requiring compatible clinical manifestations (e.g., temperature instability, respiratory distress, poor feeding, or lethargy) with at least one abnormal laboratory parameter (leukocyte count < 5000 or >25,000/mm^3^, elevated C-reactive protein, or thrombocytopenia), with or without positive blood culture confirmation. Both early-onset and late-onset sepsis cases were included if clinical and laboratory findings supported the diagnosis [17,18]. A total of 80 neonates met the inclusion criteria, consisting of 40 survivors and 40 non-survivors during their NICU admission. Cases with incomplete records, missing hematological data, or uncertain outcomes were excluded from the analysis.

### 2.3. Data Collection Procedures

Data were obtained retrospectively from the hospital’s electronic medical record system. Demographic information (sex, gestational age, birth weight), clinical variables (Apgar score, fetal distress, and maternal conditions), and laboratory parameters were extracted using a standardized data collection form. The confidentiality of all patient data was strictly maintained; no patient-identifiable information was recorded or reported.

### 2.4. Study Variables

Dependent variable: In-hospital mortality among neonates with sepsis.

Independent variables: CRIB II score, hemoglobin (Hb), NLR, PLR, RDW, mean platelet volume (MPV), and maternal factors such as preeclampsia, hypertension, urinary tract infection, chorioamnionitis, and prolonged rupture of membranes (PROM > 24 h).

### 2.5. CRIB II Scoring

The CRIB II score was calculated using five parameters: gestational age, birth weight, sex, admission temperature, and base excess within the first 12 h of life. Each variable was assigned a score according to the CRIB II scoring system, and the total score ranged from 0 to 27. Higher scores indicated greater severity and predicted mortality risk.

### 2.6. Hematological Parameters

Hematologic values were obtained from the first complete blood count (CBC) recorded within 24 h of admission. The following indices were evaluated:

NLR: ratio of absolute neutrophil count to lymphocyte count.

PLR: ratio of platelet count to lymphocyte count.

RDW and MPV: direct values from automated hematology analyzers.

### 2.7. Statistical Analysis

All data analyses were performed using IBM SPSS Statistics 25.0 The normality of continuous data was assessed using the Kolmogorov–Smirnov test. Normally distributed variables were presented as mean ± standard deviation (SD), whereas non-normally distributed data were expressed as median (minimum–maximum). Categorical variables were summarized as frequencies and percentages.

Comparisons between survivor and non-survivor groups were performed using the independent-samples *t*-test or Mann–Whitney U test for continuous variables, and the Chi-square test or Fisher’s exact test for categorical variables. Variables with a *p*-value < 0.25 in the bivariate analysis were subsequently entered into a multivariate logistic regression model to identify independent predictors of neonatal mortality. The strength of association was expressed as odds ratios (ORs) with 95% confidence intervals (CIs). Model discrimination was further evaluated using ROC curve analysis, and a *p*-value < 0.05 was considered statistically significant.

## 3. Results

### 3.1. Demographic and Clinical Characteristics

A total of 80 neonates diagnosed with sepsis were enrolled, comprising 40 survivors (50%) and 40 non-survivors (50%). The demographic and clinical characteristics of both groups are summarized in Table 1.

No significant differences were observed between survivors and non-survivors in terms of sex distribution (*p* = 1.000) or gestational age (*p* = 0.501). However, several neonatal characteristics were significantly associated with mortality. Low birth weight (<2500 g) was found in 61.9% of non-survivors compared to 38.1% of survivors (*p* = 0.025). Neonates with low Apgar scores had a markedly higher mortality rate (68.3%) than those with normal scores (30.8%) (*p* < 0.001). Fetal distress occurred more frequently among non-survivors (69.0%) than survivors (31.0%) (*p* = 0.011).

Regarding maternal characteristics, conditions such as preeclampsia, hypertension, chorioamnionitis, urinary tract infection, and other comorbidities showed no significant association with neonatal mortality (*p* > 0.05). Nevertheless, there was a trend toward higher mortality among neonates born to mothers with prolonged rupture of membranes (PROM > 24 h) (*p* = 0.070).

### 3.2. Hematological Parameters and CRIB II Score

Hematological profiles and CRIB II scores are presented in Table 2. Non-survivors exhibited significantly lower mean hemoglobin levels (12.7 ± 4.0 g/dL) than survivors (14.3 ± 2.3 g/dL) (*p* = 0.037). The median NLR and PLR were also higher among non-survivors (NLR: 2.8 vs. 1.7, *p* = 0.004; PLR: 88.5 vs. 51.8, *p* < 0.001). Similarly, RDW and MPV were significantly elevated in non-survivors (RDW: 16.8 vs. 15.7, *p* = 0.033; MPV: 10.5 vs. 9.5, *p* < 0.001).

The CRIB II score was notably higher in non-survivors (median 5.0 [1.0–13.0]) than survivors (2.0 [0–7.0]) (*p* < 0.001). Receiver operating characteristic (ROC) curve analysis demonstrated that the CRIB II score had the highest predictive accuracy for mortality (AUC = 0.835; optimal cut-off = 3.5; sensitivity 82.5%; specificity 78.0%), followed by PLR (AUC = 0.757; cut-off = 81.33; sensitivity 80.0%; specificity 70.0%) and MPV (AUC = 0.729; cut-off = 9.8 fL; sensitivity 75.0%; specificity 68.0%), indicating good discriminative performance.

### 3.3. Multivariate Analysis

Variables with *p* < 0.25 in bivariate analysis were entered into a multivariate logistic regression model to identify independent predictors of mortality (Table 3). Three variables remained statistically significant:

- Prolonged rupture of membranes (PROM > 24 h): OR = 12.23; 95% CI: 2.05–73.11; *p* = 0.006
- Platelet-to-lymphocyte ratio (PLR > 81.33): OR = 91.02; 95% CI: 7.00–1184.29; *p* = 0.001
- CRIB II score (>3.5): OR = 101.37; 95% CI: 4.50–2284.47; *p* = 0.004

The model demonstrated good calibration according to the Hosmer–Lemeshow goodness-of-fit test (χ^2^ = 6.21, *p* = 0.623), suggesting adequate model fit without evidence of overfitting. Both the CRIB II score and PLR remained strong, independent predictors of neonatal mortality, while maternal PROM represented a significant perinatal risk factor for poor outcomes.

## 4. Discussion

This study evaluated the predictive value of the CRIB II score and selected hematological markers, alongside maternal–fetal risk factors, in determining mortality among neonates with sepsis. The findings demonstrate that CRIB II > 3.5, PLR > 81.33, and PROM > 24 h were independent predictors of in-hospital mortality. These results emphasize the combined prognostic utility of a validated physiological score and simple blood-based indices in neonatal critical care.

The overall mortality rate in this cohort (50%) is comparable to previously reported figures in low-resource neonatal intensive care units (NICUs), where case-fatality rates range from 30% to 60% [2,13]. Consistent with prior research, low birth weight and low Apgar scores were significantly associated with poor outcomes, highlighting the vulnerability of preterm and growth-restricted infants to sepsis-related complications [10,12]. Although maternal comorbidities such as preeclampsia and infection were not independently related to mortality, PROM > 24 h emerged as a significant risk factor, supporting earlier findings that prolonged membrane rupture facilitates ascending intrauterine infection and neonatal colonization [14,16,19]. More recent analyses confirm that PROM beyond 18–24 h markedly increases the risk of early-onset sepsis and adverse neonatal outcomes, even with prophylactic antibiotics [18,20,21].

The present study identified a CRIB II cut-off value > 3.5 as a strong predictor of mortality—slightly lower than thresholds reported in other studies (5–6) [8,10]. This variation may reflect population differences, including a higher proportion of late-onset sepsis and term neonates in the current cohort. Previous multicenter studies have validated the CRIB II score as a reliable prognostic tool in preterm and critically ill infants, demonstrating strong correlation with survival outcomes (AUC 0.80–0.90) [8,9,10]. However, evidence specifically addressing septic neonates remains limited. Our findings extend this evidence by demonstrating that CRIB II maintains good discriminative performance (AUC = 0.835) even in infection-driven critical illness, suggesting its usefulness beyond the original population for which it was designed.

In addition to CRIB II, hematologic indices—particularly the PLR and MPV—were associated with mortality. Elevated PLR reflects both thrombocytopenia and lymphopenia, indicative of systemic inflammation and immune dysregulation in sepsis [11,12]. The prognostic significance of MPV observed in this study aligns with earlier evidence suggesting that platelet activation contributes to microvascular injury and organ dysfunction in neonatal and pediatric sepsis [22,23]. Our results corroborate those of Rana et al. [11] and J et al. [12], who also reported higher NLR and PLR values in septic neonates, but we further demonstrate their independent association with mortality when combined with a physiological score. This integrated model may provide clinicians with an early, accessible, and objective tool for mortality risk stratification—particularly valuable in LMICs where advanced biomarkers such as procalcitonin or cytokine assays are not routinely available.

The multivariate model in our study showed good calibration by the Hosmer–Lemeshow test (*p* = 0.623), indicating adequate model fit and internal validity despite the modest sample size. Nevertheless, future studies with larger multicenter cohorts should perform external validation and explore dynamic scoring systems such as nSOFA for comparison [10]. It is also important to differentiate between early- and late-onset sepsis in future analyses, as their microbial etiologies and inflammatory responses differ significantly [5,6].

Finally, our findings contribute to the broader public-health agenda of reducing preventable neonatal deaths in line with the United Nations Sustainable Development Goal (SDG) 3.2, which aims to end neonatal mortality from avoidable causes by 2030 [24]. By integrating simple hematologic markers and CRIB II scoring into early sepsis evaluation, healthcare providers in resource-limited settings can improve triage decisions and prioritize intensive monitoring for high-risk neonates.

### 4.1. Clinical and Policy Implications

From a clinical perspective, the combination of the CRIB II score with hematologic indices such as PLR offers a practical and inexpensive approach to early mortality risk stratification. Because these parameters are routinely available at admission, their use could facilitate rapid identification of high-risk neonates, enabling early intervention and allocation of intensive care resources. From a policy standpoint, incorporating CRIB II and hematologic markers into national neonatal care protocols could improve the uniformity and quality of sepsis management, particularly in low- and middle-income countries. This approach also aligns with global initiatives such as Sustainable Development Goal 3.2, which targets the reduction in preventable neonatal deaths.

### 4.2. Strengths and Limitations

The main strength of this study lies in its integration of a validated clinical score with objective, inexpensive laboratory biomarkers, providing a cost-effective prognostic model applicable in routine NICU settings. However, certain limitations must be acknowledged. First, the single-center design and relatively small sample size may limit generalizability. Second, as a retrospective analysis, it was dependent on the accuracy and completeness of recorded data. Third, external validation across multiple centers and different patient populations would strengthen the reliability of the predictive model.

### 4.3. Future Directions

Future research should focus on prospective, multicenter validation of the combined CRIB II–PLR model, potentially incorporating additional biomarkers such as C-reactive protein (CRP) or procalcitonin to enhance prognostic accuracy. Implementation studies evaluating the clinical impact of risk-based management algorithms on neonatal outcomes would also provide valuable insights.

## 5. Conclusions

In this study, the CRIB II score and PLR emerged as strong, independent predictors of in-hospital mortality among neonates with sepsis, while PROM was identified as a significant maternal risk factor.

The combination of a validated clinical severity score with simple hematological biomarkers provides a practical, objective, and cost-effective approach for early risk stratification in neonatal sepsis. This integrative model highlights the value of combining clinical and laboratory data to improve prognostic accuracy and guide timely, targeted management strategies.

Further multicenter prospective studies are warranted to validate these findings and refine predictive models that can be broadly implemented across diverse healthcare settings.

## Figures and Tables

**Table 1:** Demographic and clinical characteristics of neonates with sepsis.

Demographic and Clinical Characteristics	Non-Survivor	Survivor	Total	*p*-Value
Sex Distribution				1.000
Male	18 (48.6)	19 (51.4)	37 (46.3)	
Female	22 (51.2)	21 (51.2)	43 (53.8)	
Gestational Age				
Premature (24–36 Weeks)	20 (54.1)	17 (45.9)	37 (46.3)	0.501
Aterm (37–40 Weeks)	20 (46.5)	23 (53.5)	43 (53.8)	
Birth Weight				0.025 *
Low Birth Weight (LBW)	26 (61.9)	16 (38.1)	42 (52.5)	
Normal Birth Weight	14 (36.8)	24 (63.2)	38 (47.5)	
APGAR Scores				<0.001 *
Low APGAR Scores	28 (68.3)	13 (31.7)	41 (51.3)	
Normal APGAR Scores	12 (30.8)	27 (69.2)	39 (48.8)	
Fetal Distress				0.011 *
Yes	20 (69.0)	9 (31.0)	29 (36.3)	
No	20 (39.2)	31 (60.8)	51 (63.8)	
Maternal Factors				0.614
Preeclampsia	5 (45.5)	6 (54.5)	11 (13.8)	
Placenta Accreta	14 (66.7)	7 (33.3)	21 (26.3)	
Oligohydramnios/Anhydramnios	9 (52.9)	8 (47.1)	17 (21.3)	
Hypertension	3 (33.3)	6 (66.7)	9 (11.3)	
Chorioamnionitis	1 (20.0)	4 (80.0)	5 (6.3)	
Maternal Diabetes Melitus (DM)	3 (50.0)	3 (50.0)	6 (7.5)	
Urinary Tract Infection (UTI)	3 (50.0)	3 (50.0)	6 (7.5)	
Maternal Vaginal Discharge	2 (40.0)	3 (60.0)	5 (6.3)	
History of Premature Rupture of Membranes (PROM)				0.007 *
>24 h	24 (61.5)	15 (38.5)	39 (48.8)	
6–24 h	10 (47.6)	11 (52.4)	21 (26.3)	
<6 h	6 (30.0)	14 (70.0)	20 (25.0)	
History of Maternal Fever				0.499
Yes	4 (40.0)	6 (60.0)	10 (12.5)	
No	36 (51.4)	34 (48.6)	70 (87.5)	
History of Foul-Smelling Amniotic Fluid				0.675
Yes	2 (33.3)	4 (66.7)	6 (7.5)	
No	38 (51.4)	36 (48.6)	74 (92.5)	
Multiple Pregnancy				1.000
Yes	3 (42.9)	4 (57.1)	7 (8.8)	
No	37 (50.7)	36 (49.3)	73 (91.3)	

* *p* < 0.05.

**Table 2:** Comparison of hematological parameters and CRIB II score between survivors and non-survivors.

Hematological Parameters	Non-Survivor	Survivor	*p*-Value	AUC (Area Under the Curve)
Hemoglobin, Mean	12.7 ± 4.0	14.3 ± 2.3	0.037 *	0.345
Leucocytes, Median (Min–Max)	15.065 (3.850–99.770)	13.910 (1.360–40.170)	0.773	0.519
Platelet, Median (Min–Max)	219.000(12.000–610.000)	202.000 (38.000–868.000)	0.356	0.560
ANC, Median (Min–Max)	8975.7 (509.9–91.489.1)	7.172 (470.6–29.083.1)	0.780	0.518
NLR, (Min–Max)	2.8 (0.3–32.8)	1.7 (0.6–11.2)	0.004 *	0.686
PLR, Median (Min–Max)	88.5 (2.3–606.8)	51.8 (14.2–95.5)	<0.001 *	0.757
RDW, Median (Min–Max)	16.8 (13.7–21.6)	15.7 (13.8–21.0)	0.033 *	0.638
MPV, Median (Min–Max)	10.5 (7.9–13.8)	9.5 (8.2–12.4)	<0.001 *	0.729
CRIB-II, Median (Min–Max)	5.0 (1.0–13.0)	2.0 (0–7.0)	<0.001 *	0.835

* *p* < 0.05; ANC, absolute neutrophil count; NLR, neutrophil-to-lymphocyte ratio; PLR, platelet-to-lymphocyte ratio; RDW, red cell distribution width; MPV, mean platelet volume; CRIB II, Clinical Risk Index for Babies II.

**Table 3:** Multivariate Analysis of Predictors of Mortality.

Variable	Multivariate Analysis		
	OR	95% CI	*p* Value
History of PROM	12.23	2.05–73.11	0.006 *
PLR Scores > 81.33	9.102	7.00–1184.29	0.001 *
CRIB-II > 3.50	10.137	4.50–2284.47	0.004 *

* *p* < 0.05; PROM, premature rupture of membranes.

## Data Availability

All data supporting the findings of this study are included within the article. Additional data can be provided by the corresponding author upon reasonable request.
